# Kinetic Models for Epidemic Dynamics in the Presence of Opinion Polarization

**DOI:** 10.1007/s11538-023-01147-2

**Published:** 2023-03-29

**Authors:** Mattia Zanella

**Affiliations:** Department of Mathematics “F. Casorati”, University of Pavia, Pavia, Italy

**Keywords:** Kinetic equations, Mathematical epidemiology, Opinion dynamics, 92D30, 35Q20, 35Q84, 35Q92

## Abstract

Understanding the impact of collective social phenomena in epidemic dynamics is a crucial task to effectively contain the disease spread. In this work, we build a mathematical description for assessing the interplay between opinion polarization and the evolution of a disease. The proposed kinetic approach describes the evolution of aggregate quantities characterizing the agents belonging to epidemiologically relevant states and will show that the spread of the disease is closely related to consensus dynamics distribution in which opinion polarization may emerge. In the present modelling framework, microscopic consensus formation dynamics can be linked to macroscopic epidemic trends to trigger the collective adherence to protective measures. We conduct numerical investigations which confirm the ability of the model to describe different phenomena related to the spread of an epidemic.

## Introduction

During the outbreak of SARS-CoV-2 pandemic, we observed how, as cases escalated, collective compliance to the so-called non-pharmaceutical interventions (NPIs) was crucial to ensure public health in the absence of effective treatments, see, e.g. Albi et al. (2021), Bellomo and Chaplain (2022), Bertaglia et al. (2021), Gatto et al. (2020), Viguerie et al. (2021) and Zanella et al. (2021). Nevertheless, the effectiveness of lockdown measures heavily depended on the beliefs/opinions of individuals regarding protective behaviour, which are thus linked to personal situational awareness (Durham and Casman 2012; Tchuenche et al. 2011). Recent experimental results have shown that social norm changes are often triggered by opinion alignment phenomena (Tunçgenç et al. 2021). In particular, the perceived adherence of individuals’ social network has a strong impact on the effective support of the protective behaviour. The individual responses to threat are a core question to set up effective measures prescribing norm changes in daily social contacts (Dezecache et al. 2020), and cases escalation is a factor that may be perceived in different ways. For these reasons, it appears natural to couple classical epidemiological models with opinion dynamics in order to understand the mutual influence of these phenomena.

In recent years, the study of emerging properties of large systems of agents has obtained a growing interest in heterogeneous communities in social and life sciences, see, e.g. Bolley et al. (2011), Barré et al. (2017), Carrillo et al. (2010a, 2010b), Chalub et al. (2004), Cordier et al. (2005), Ciallella et al. (2021), Degond and Motsch (2008), Fornasier et al. (2011), Ha and Tadmor (2008) and Motsch and Tadmor (2014). In particular, thanks to their cooperative nature, the dynamics leading to opinion formation phenomena have been often described through the methods of statistical mechanics (Ben-Naim et al. 2003; Castellano et al. 2009; Hegselmann and Krause 2002; Sznajd-Weron and Sznajd 2000; Weidlich 2000). Among other approaches, kinetic theory provided a sound theoretical framework to investigate the emerging patterns of such systems (Düring et al. 2009; Düring and Wolfram 2015; Toscani 2006). In this modelling setting, the microscopic, individual-based, opinion variations take place through binary interaction schemes involving the presence of social forces, whose effects are observable at the macroscopic scale (Pareschi et al. 2019). The equilibrium distribution describes the formation of a relative consensus about certain opinions (Pareschi and Toscani 2013; Toscani 2006; Toscani et al. 2018). In this direction, it is of paramount importance to obtain reduced complexity models whose equilibrium distribution is explicitly available under minimal assumptions (Furioli et al. 2019; Toscani 2006). The deviation from global consensus appears in the form of opinion polarization, i.e. the divergence away from central positions towards extremes (Loy et al. 2022). This latter feature of the agents’ opinion distribution is frequently observed in problems of choice formation (Aletti et al. 2007).

The derivation of classical compartmental epidemiological dynamics from particle systems has been recently explored as a follow-up question on the effectiveness of available modelling approaches. Indeed, epidemics, as well as many other collective phenomena, can be easily thought as a result of repeated interactions between a large number of individuals that eventually modify their epidemiological state. The transition rates between epidemiologically relevant states are furthermore influenced by several phenomena linked to the disease itself and to the social behaviour of individuals. Without attempting to revise the whole literature, we mention Albi et al. (2022), Bertaglia et al. (2021), Della Marca et al. (2022a), Dimarco et al. (2020, 2021, 2022), Loy and Tosin (2021) and the references therein for an introduction to the subject. Among them, contact dynamics are particularly relevant for contact-based disease transmissions.

In this work, we introduce a novel kinetic model that takes into account opinion formation dynamics of the individuals’ protective behaviour coupled with epidemic spreading. These dynamics will result structurally linked due to the mutual influence of opinion formation processes and the transmission of the infection. The effects of behavioural dynamics on epidemic models have been investigated at the population level, see Poletti et al. (2009). In particular, the formation of opinion clustering is connected to vaccination hesitancy, see, e.g. Buonomo et al. (2022) and the references therein. In this direction, we mention the recent results in Della Marca et al. (2022b), Giambiagi Ferrari et al. (2021), Kontorovsky et al. (2022) and Zhou et al. (2019) where agent-based dynamics are upscaled at the level of observable epidemiological quantities.

Kinetic equations are capable of providing efficient methods to bridge the microscopic, often unobservable, scale of individual agents, where elementary fundamental dynamics take place, and the macroscopic scale of observable manifestations. Indeed, in classical kinetic theory, the possibility to derive hydrodynamic descriptions of particles’ systems is of paramount importance for providing real-time predictions. In the context of multiagent systems, the problem of deriving macroscopic equations is underexplored and has to face additional challenges in the definition of the social forces involved in the interactions. In order to get analytical insights on the macroscopic behaviour of the system, the derivation of reduced complexity models is a key point. Hence, thanks to the derived surrogate models we can derive equilibrium profiles that are coherent with the ones defined at the kinetic level. In this work, we exploit the Fokker–Planck modelling approach that has been introduced in Toscani (2006) for opinion formation processes. We remark that, at variance with Dimarco et al. (2021, 2022), the interactions between agents are structurally binary to mimic compromise behaviour. The new derived macroscopic models encode all the information of the opinion-based interactions and describe coherent transition rates penalizing agents clustering on a weak protective behaviour. We will observe how opinion polarization can trigger an increasing spread of infection in society.

In more details, the paper is organized as follows: in Sect. 2, we introduce a kinetic epidemic model where agents are characterized by their epidemiological state and their opinion. Hence, a reduced complexity operator is derived to compute the large time opinion distribution of the system of agents and we discuss minimal assumptions to observe opinion polarization. In Sect. 3, we derive a macroscopic system of equations by considering an equilibrium closure method. The derived macroscopic model expresses the evolution at the epidemic scale of the conserved quantities in the operator for opinion exchanges. Finally, in Sect. 4 we present several numerical tests showing the coherence of the presented closure strategy with the initial kinetic model in suitable scales. Furthermore, in the latter section we explore the possibility of considering more complex interaction functions in the opinion exchange process together with the influence of opinion polarization on the spreading of the disease.

## A Kinetic Model Approach for Consensus Formation and Epidemic Dynamics

In this section, we introduce a kinetic compartmental model for the spreading of an infectious disease that is coupled with the evolution of the opinions of individuals. We consider a system of agents that can be subdivided in the following epidemiologically relevant states: susceptible (*S*) agents are the ones that can contract the disease, infectious agents (*I*) are responsible for the spread of the disease, exposed (*E*) agents have been infected but are still not contagious and, finally, removed (*R*) agents cannot spread the disease. Each agent is endowed of a continuous opinion variable $w \in I$ which varies continuously in $I = [-1,1]$, where $-1$ and 1 denote two opposite beliefs on the protective behaviour. In particular, $w = -1$ means that the agents do not believe in the necessity of protections (like wearing masks or reducing daily contacts), whereas $w= 1$ is linked to maximal agreement on protective behaviour. We also assume that agents characterized by high protective behaviour are less likely to contract the infection.

With the aim to incorporate the impact of opinion evolution in the dynamics of infection, we denote by $f_J(w,t)$ the distribution of opinions at time $t \ge 0$ of agents in the compartment $J \in \mathcal C = \{S,E,I,R\}$. In particular, $f_J = f_J(w,t):[-1,1] \times \mathbb R_+ \rightarrow \mathbb R_+$ is such that $f_J(w,t)dw$ represents the fraction of agents with opinion in $[w,w+dw]$ at time $t\ge 0$ in the *J*th compartment. Furthermore, we impose

$$\begin{aligned} \sum _{J \in \mathcal C} f_J(w,t) = f(w,t), \qquad \int _{-1}^{1} f(w,t)\hbox {d}w = 1, \end{aligned}$$

while the mass fractions of the population in each compartment and their moment of order $r>0$ are given by

1 $$\begin{aligned} \rho _J(t) = \int _{-1}^1 f_J(w,t)\hbox {d}w, \qquad \rho _J(w,t) m_{r,J} = \int _{-1}^1 w^r f_J(w,t)\hbox {d}w. \end{aligned}$$

In the following, to simplify notations, we will indicate with $m_J(t)$, $J \in \mathcal C$, the mean opinion in the compartment *J* corresponding to $r = 1$.

**Fig. 1:**
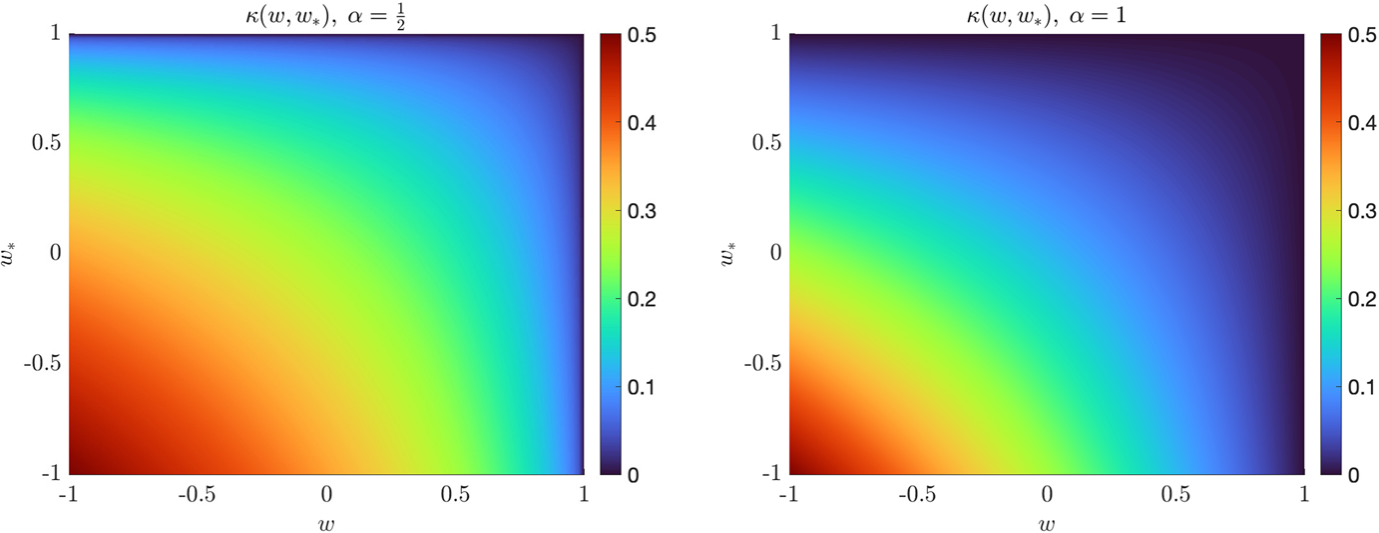
(Color figure online) We sketch the function $\kappa (w,w_*)$ in (4) for $\alpha =\frac{1}{2}$ (left) and $\alpha = 1$ (right). In both cases, we fixed the coefficient $\beta = \frac{1}{2}$

We assume that the introduced compartments of the model can have different impact in the opinion dynamics. The kinetic model for the coupled evolution of opinions and infection is given by the following system of kinetic equations

2 $$\begin{aligned} \begin{aligned} \partial _t f_S(w,t)&= -K(f_S,f_I)(w,t) + \dfrac{1}{\tau } Q_{S}(f_S,f_S)(w,t), \\ \partial _t f_E(w,t)&= K(f_S,f_I)(w,t) - \sigma _E f_E(w,t) + \dfrac{1}{\tau }Q_{E}(f_E,f_E)(w,t), \\ \partial _t f_I(w,t)&= \sigma _E f_E(w,t) - \gamma f_I(w,t) + \dfrac{1}{\tau } Q_{I}(f_I,f_I)(w,t), \\ \partial _t f_R(w,t)&= \gamma f_I(w,t) + \dfrac{1}{\tau }Q_{R}(f_R,f_R)(w,t), \end{aligned} \end{aligned}$$

where $\tau >0$ and $Q_{J}(\cdot ,\cdot )$ characterizes the evolution of opinions of agents that belong to the compartment $J \in \mathcal C$. In the next section, we will specify the form of these operators describing binary opinion interactions among agents. The parameter $\sigma _E>0$ is such that $1/\sigma _E$ measures the mean latent period for the disease, whereas $\gamma >0$ is such that $1/\gamma >0$ is the mean infectious period (Diekmann and Heesterbeek 2000). In (2), the transmission of the infection is governed by the local incidence rate

3 $$\begin{aligned} K(f_S,f_I)(w,t) = f_S(w,t) \int _{-1}^1 \kappa (w,w_*)f_I(w_*,t)\hbox {d}w_*, \end{aligned}$$

where $\kappa (w,w_*)$ is a nonnegative decreasing function measuring the impact of the protective behaviour among different compartments. A leading example for the function $\kappa (w,w_*)$ can be obtained by assuming

4 $$\begin{aligned} \kappa (w,w_*) = \dfrac{\beta }{4^{\alpha }} (1-w)^{\alpha }(1-w_*)^{\alpha }, \end{aligned}$$

where $\beta >0$ is the baseline transmission rate characterizing the epidemics and $\alpha >0$ is a coefficient linked to the efficacy of the protective measures. In Fig. 1, we represent the introduced function $\kappa (\cdot ,\cdot )$ for several values of $\alpha >0$. We may observe how for $\alpha \equiv 0$ the influence of opinion dynamics on the epidemiological model disappears. We highlight that in the simple case $\alpha = 1$ we get

$$\begin{aligned} K(f_S,f_I)(w,t) = \dfrac{\beta }{4}(1-w)f_S(w,t)(1-m_I(t))I(t)\ge 0, \qquad I(t)\ge 0 \end{aligned}$$

with $K(f_S,f_I) \equiv 0$ in the case $m_I\equiv 1$ or in the case where all susceptible agents are concentrated in the maximal protective behaviour $w = 1$.

### Kinetic Models for Opinion Formation

The dynamics of opinion formation have often been described by resorting to methods of statistical physics, see, e.g. Castellano et al. (2009) and Galam (1997). In particular, kinetic theory provides a sound theoretical background to model fundamental interactions among agents and to provide a convenient dynamical structure for related follow-up questions on control problems and network formation (Albi et al. 2017; Toscani 2006). In the aforementioned kinetic models, the opinion variation of large systems of agents depends on binary interactions whose are driven by social forces determining the formation of consensus about certain opinions. The emerging distribution of opinions can be evaluated at the macroscopic level (Motsch and Tadmor 2014; Pareschi and Toscani 2013). Recent advancements have been devoted to include external influences in opinion formation models to capture realistic complex phenomena. Without intending to review the very huge literature on the topic, we mention Ben-Naim et al. (2003), Cristiani and Tosin (2018), Düring et al. (2009), Düring and Wolfram (2015) and the references therein.

The elementary interactions between agents weight two opposite behaviours: the first is the compromise propensity, i.e. the tendency to reduce the opinion distance after interaction, and the second is the self-thinking, corresponding to unpredictable opinion deviations. In details, an interaction between two individuals in the compartments $J \in \mathcal C$ with opinion pair $(w,w_*)$ leads to an opinion pair $(w^{\prime }, w^{\prime }_*)$ defined by the relations

5 $$\begin{aligned} \begin{aligned} w^\prime&= w + \lambda _J P(w,w_*) (w_*-w) + D(w)\eta _{J} \\ w^\prime _*&= w_* + \lambda _J P(w_*,w)(w-w_*) + D(w_*)\tilde{\eta }_{J}, \end{aligned} \end{aligned}$$

where $\lambda _J \in (0,1)$ and $P(w,w_*) \in [0,1]$ is an interaction function. In (5), we further introduce the local diffusion function *D*(*w*), and $\eta _{J},\tilde{\eta }_{J}$ are independent and identically distributed centred random variables with finite variance $\left\langle \eta _{J} \right\rangle = \left\langle \eta _{J} \right\rangle = \sigma _J^2$, where we indicate with $\left\langle \cdot \right\rangle$ the expected value with respect to the distribution of the random variables.

As observed in Pareschi et al. (2019), we have that the mean opinion is conserved for symmetric interaction functions, $P(w,w_*) = P(w_*,w)$ for all $w,w_* \in [-1,1]$. Indeed, from (5) we get

6 $$\begin{aligned} \left\langle w^\prime + w_*^\prime \right\rangle = w+w_* +\lambda _J( P (w,w_*) - P(w_*,w) )(w_*-w), \end{aligned}$$

which reduces to $\left\langle w^\prime + w_*^\prime \right\rangle = w+w_*$ under the aforementioned assumptions. Furthermore, if we consider the mean energy, we get

$$\begin{aligned} \begin{aligned} \left\langle (w^\prime )^2 + (w^\prime _*)^2 \right\rangle =&\, w^2 + w_*^2 +\lambda _J^2\left[ P^2(w,w_*) +P^2(w_*,w)\right] (w_*-w)^2 \\&+2\lambda _J[ P(w,w_*)w - P(w_*,w)w_*](w_*-w)\\&+ \sigma _J^2(D^2(w)+D^2(w_*)), \end{aligned} \end{aligned}$$

meaning that the energy is not conserved on average in a single binary interaction. In the absence of the stochastic component, $\sigma _J^2\equiv 0$, we get that for symmetric interactions the mean energy is dissipated

$$\begin{aligned} \left\langle (w^\prime )^2 + (w^\prime _{*})^2 \right\rangle = w^2 + w_*^2 - 2\lambda _J P(w,w_*)(w_*-w)^2 + o(\lambda _J)\le w^2 + w_*^2 + o(\lambda _J) \end{aligned}$$

The physical admissibility of interaction rules (5) is provided if $|w_{}^\prime |, |w_{*}^\prime | \le 1$ for $|w|,|w_*| \le 1$. We observe that

$$\begin{aligned} \begin{aligned} |w_{}^\prime |&\le |(1-\lambda _JP(w,w_*))w + \lambda _J P(w,w_*)w_* + D(w)\eta _J| \\&\le (1-\lambda _J P(w,w_*))|w|+\lambda _J P(w,w_*) + D(w)|\eta _J|, \end{aligned}\end{aligned}$$

since $|w_*| \le 1$, from which we get that the sufficient condition for $|w_{}^\prime | \le 1$ is provided by

$$\begin{aligned} D(w)|\eta _{J}| \le (1-\lambda _J P(w,w_*))(1-|w|), \end{aligned}$$

which is satisfied if a constant $$c>0 $$ exists and is such that

7 $$\begin{aligned} {\left\{ \begin{array}{ll} |\eta _{J}| \le c(1-\lambda _J P(w,w_*)) \\ c \cdot D(w) \le 1-|w|, \end{array}\right. } \end{aligned}$$

for all $w,w_* \in [-1,1]$. Since $0\le P(\cdot ,\cdot )\le 1$ by assumption, the first condition in (7) can be enforced by requiring that

$$\begin{aligned} |\eta _{J}| \le c(1-\lambda _J). \end{aligned}$$

Therefore, it is sufficient to consider the support of the random variables determined by $|\eta _J| \le c(1-\lambda _J)$. The second condition in (7) forces $D(\pm 1)=0$. Other choices for the local diffusion function have been investigated in Pareschi et al. (2019) and Toscani (2006).

The collective trends of a system of agents undergoing binary interactions (5) are determined by a Boltzmann-type model having the form

8 $$\begin{aligned} \partial _t f_J(w,t) = \dfrac{1}{\tau } Q_{J}(f_J,f_J), \end{aligned}$$

with $$\tau >0$$ and

$$\begin{aligned} Q_{J}(f_J,f_J)(w,t) = \left\langle \int _{-1}^1 \left( \dfrac{1}{{}^\prime \mathcal J} f_J({}^\prime w,t)f_J({}^\prime w_*,t)-f_J(w,t)f_J(w_*,t) \right) \hbox {d}w_* \right\rangle , \end{aligned}$$

where $({}^\prime w,{}^\prime w_*)$ are pre-interaction opinions generating the post-interaction opinions $(w,w_*)$ and ${}^\prime \mathcal J$ is the Jacobian of the transformation $({}^\prime w,{}^\prime w_*) \rightarrow (w,w_*)$.

### Derivation of a Fokker–Planck Model

The equilibrium distribution of the kinetic model (8) is very difficult to obtain analytically. For this reason, several reduced complexity models have been proposed. In this direction, a deeper insight on the equilibrium distribution of the kinetic model can be obtained by introducing a rescaling of both the interaction and diffusion parameters having roots in the so-called grazing collision limit of the classical Boltzmann equation (Cercignani 1988; Pareschi and Toscani 2013). The resulting model has the form of an aggregation–diffusion Fokker–Planck-type equation, encapsulating the information of microscopic dynamics. For the obtained surrogate model, the study of asymptotic properties is typically easier than the original kinetic model.

We start by observing that we can conveniently express the operators $Q_{J}(\cdot ,\cdot )$ in weak form. Let $\varphi (w)$ denote a test function; thus, for $J \in \mathcal C$ we have

$$\begin{aligned} \begin{aligned}&\int _{-1}^1 \varphi (w)Q_{J}(f_J,f_J)(w,t)\hbox {d}w \\&\qquad = \left\langle \int _{-1}^1 (\varphi (w^\prime ) - \varphi (w)) f_J(w,t)f_J(w,t)\hbox {d}w_*\,\hbox {d}w \right\rangle , \end{aligned}\end{aligned}$$

where $w^\prime$ is defined in (5). The prototype of a symmetric interaction function *P* is given by the constant function $P\equiv 1$. In this case, we may obtain analytic insight on the large time distribution of the system by resorting to a reduced complexity Fokker–Planck-type model (Toscani 2006). We introduce the so-called quasi-invariant regime

9 $$\begin{aligned} \lambda _J \rightarrow \epsilon \lambda _J,\qquad \sigma _J^2 \rightarrow \epsilon \sigma _J^2, \end{aligned}$$

where $\epsilon >0$ is a scaling coefficient. We have

$$\begin{aligned} \begin{aligned}&\varphi (w^\prime ) - \varphi (w) \\&\quad = \varphi ^\prime (w)\left\langle w^\prime -w \right\rangle + \dfrac{1}{2}\varphi ^{\prime \prime }(w) \left\langle (w^\prime _{}-w)^2 \right\rangle + \dfrac{1}{6}\varphi ^{\prime \prime \prime }(\bar{w}_{})\left\langle (w^\prime _{}-w)^3 \right\rangle , \end{aligned} \end{aligned}$$

where $\min \{w,w_{}^\prime \}< \bar{w}_{}< \max \{w,w_{}^\prime \}$. Plugging the above expansions in the Boltzmann-type model, we have

10 $$\begin{aligned} \begin{aligned}&\dfrac{\hbox {d}}{\hbox {d}t} \int _{-1}^1 \varphi (w)f_J(w,t)\hbox {d}w = \\&\qquad \epsilon \lambda _J \rho _J \int _{-1}^1 \int _{-1}^1\varphi ^\prime (w)(m_J-w)f_J(w,t)\hbox {d}w \\&\qquad + \dfrac{\epsilon \sigma ^2}{2}\int _{-1}^1 \varphi ^{\prime \prime }(w)D^2(w)f_J(w,t)\hbox {d}w + R(f_J,f_J), \end{aligned} \end{aligned}$$

where $R(f_J,f_J)$ is a reminder term

$$\begin{aligned} \begin{aligned}&R(f_J,f_J)(w,t) = \dfrac{1}{2} \int _{-1}^{1}\varphi ^{\prime \prime }(x)\epsilon ^2\lambda ^2_J(w_*-w)^2 f_J(w,t)\hbox {d}w \\&\quad + \dfrac{1}{6}\left\langle \int _{-1}^1\int _{-1}^1 \varphi ^{\prime \prime \prime }(w)(\epsilon \lambda _J(w_*-w) + D(w)\eta _{J})^3 f_J(w,t)f_J(w_*,t)\hbox {d}w\,\hbox {d}w_*\right\rangle \end{aligned} \end{aligned}$$

Hence, in the time scale $\xi = \epsilon t$, introducing the distribution $g_J(w,\xi ) = f_J(w,\xi /\epsilon )$, we have that $\partial _\xi g_J(w,\xi ) = \frac{1}{\epsilon } \partial _t f_J$ and (10) becomes

$$\begin{aligned} \begin{aligned}&\dfrac{\hbox {d}}{\hbox {d}\xi }\int _{-1}^1 \varphi (w)g_J(w,\xi )\hbox {d}w = \lambda _J \int _{-1}^1 \int _{-1}^1 \varphi ^\prime (w)(m_J-w)g_J(w,\xi )\hbox {d}w \\&\qquad + \dfrac{\sigma _J^2}{2}\int _{-1}^1\varphi ^{\prime \prime }(w)D^2(w)g_J(w,\xi )\hbox {d}w + \dfrac{1}{\epsilon }R(g_J,g_J)(w,\xi ), \end{aligned} \end{aligned}$$

where now $\frac{1}{\epsilon }R(g_J,g_J) \rightarrow 0$ under the additional hypothesis $\left\langle |\eta _{J}|^3 \right\rangle <+\infty$, see Cordier et al. (2005) and Toscani (2006). Consequently, for $\epsilon \rightarrow 0^+$, from the above equation we have

$$\begin{aligned} \begin{aligned} \dfrac{\hbox {d}}{\hbox {d}\xi }\int _{-1}^1 \varphi (w)g_J(w,\xi )\hbox {d}w =&\, \lambda _J \int _{-1}^1 \int _{-1}^1 \varphi ^\prime (w)(m_J-w)g_J(w,\xi )\hbox {d}w \\&+ \dfrac{\sigma _J^2}{2}\int _{-1}^1\varphi ^{\prime \prime }D^2(w)g_J(w,\xi )\hbox {d}w. \end{aligned}\end{aligned}$$

Now, with a slight abuse of notation, we restore $t\ge 0$ as time variable and $f_J$ as distribution. In view of the smoothness of $\varphi$, integrating back by parts the terms on the right hand side, we finally get the Fokker–Planck-type model

11 $$\begin{aligned} \begin{aligned} \partial _t f_J(w,t)&= \bar{Q}_J(f_J,f_J)(w,t) \\&= \partial _w \left[ \lambda _J(w- m_J)f_J(w,t) + \dfrac{\sigma _J^2}{2}\partial _w (D^2(w)f_J(w,t)) \right] \end{aligned} \end{aligned}$$

complemented by the following no-flux boundary conditions

$$\begin{aligned} \begin{aligned}&\lambda _J(w-m_J)f_J(w,t) + \dfrac{\sigma _J^2}{2}\partial _w (D^2(w)f_J(w,t)) \Big |_{w = \pm 1} = 0 \\&D^2(w)f_J(w,t) \Big |_{w = \pm 1 } = 0. \end{aligned}\end{aligned}$$

We can observe that the steady state of the Fokker–Planck-type model (11) is analytically computable under suitable hypotheses on the local diffusion function. If $D(w) = \sqrt{1-w^2}$, then the large time behaviour of the model is given by a beta distribution having the form

12 $$\begin{aligned} f_J^\infty (w) = \dfrac{(1+w)^{\frac{1+m_J}{\nu _J}-1} (1-w)^{\frac{1- m_J}{\nu _J}-1}}{2^{\frac{2}{\nu _J}-1}B\left( \frac{1+ m_J}{\nu _J},\frac{1- m_J}{\nu _J} \right) }, \quad \nu _J =\dfrac{ \sigma _J^2}{\lambda _J}, \end{aligned}$$

where $B(\cdot ,\cdot )$ indicates the beta function. It is worth to highlight that the first two moments of the obtained beta distribution are defined as follows:

13 $$\begin{aligned} \int _{-1}^1 w f_J^\infty (w)\hbox {d}w = m_J;\qquad \int _{-1}^1 w^2 f^\infty _J(w)\hbox {d}w = \dfrac{\nu _J}{2+\nu _J} + \dfrac{2}{2+\nu _J} m_J^2. \end{aligned}$$

**Fig. 2:**
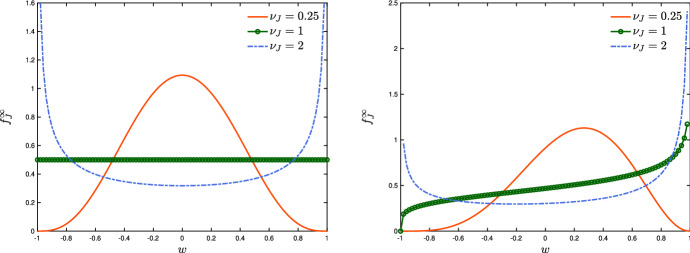
We depict the equilibrium distribution (12) for several choices of the parameter $$\nu _J>0$$ and for $$ m_J = 0$$ (left) or $$ m_J = 0.2$$ (right). Opinion polarization is observed for $$\nu _J>1$$, whereas consensus formation corresponds to $$\nu _J\ll 1$$

We can observe that the obtained model is suitable to describe classical consensus-type dynamics. This behaviour is observed if the compromise force is stronger than the one characterizing self-thinking, i.e. $$\sigma _J^2<\lambda _J$$. On the other hand, if self-thinking is stronger than the compromise propensity, i.e. $$\sigma _J^2>\lambda _J$$, we observe opinion polarization of the society. In Fig. 2, we depict the equilibrium distribution (12) for several choices of the parameter $$\nu _J>0$$. In the right figure, we assume that $$ m_J = 0$$, whereas, in the left figure, we consider the asymmetric case with $$ m_J = 0.2$$. We may observe that opinion polarization is obtained in the case $$\nu _J>1$$ as discussed.

#### Remark 1

In the more general case where interaction between agents is weighted by a nonconstant function $P(w,w_*) \in [0,1]$, we may obtain the nonlocal Fokker–Planck-type model

$$\begin{aligned} \partial _t f_J(w,t) = \partial _w \left[ \mathcal B[f_J](w,t) f_J(w,t) + \dfrac{\sigma ^2}{2}\partial _w f_J(w,t)\right] \end{aligned}$$

where

$$\begin{aligned} \mathcal B[f_J](w,t)= \int _{-1}^1 P(w,w_*)(w-w_*)f_J(w_*,t)\hbox {d}w_*. \end{aligned}$$

In this case, it is difficult to get an analytical formulation of the steady state distribution.

## Macroscopic Opinion-Based SEIR Dynamics

Once the equilibrium distribution of the operators $\bar{Q}_J(f_J,f_J)(w,t)$ is characterized, we can study the behaviour of the original system (2). In this section, we compute the evolution of observable macroscopic equations of the introduced kinetic model for epidemic dynamics with opinion-based incidence rate.

### Derivation of Moment-Based Systems

Let us rewrite the original model (2) with the reduced complexity Fokker–Planck-type operators defined in Sect. 2.2. We obtain the following model

14 $$\begin{aligned} \begin{aligned} \partial _t f_S(w,t)&= -K(f_S,f_I) + \dfrac{1}{\tau }\bar{Q}_{S}(f_S,f_S)(w,t), \\ \partial _t f_E(w,t)&= K(f_S,f_I) -\sigma _E f_E(w,t) + \dfrac{1}{\tau }\bar{Q}_E(f_E,f_E)(w,t), \\ \partial _t f_I(w,t)&= \sigma _E f_E(w,t)-\gamma f_I(w,t) + \dfrac{1}{\tau }\bar{Q}_{I}(f_I,f_I)(w,t), \\ \partial _t f_R(w,t )&= \gamma f_I(w,t) + \dfrac{1}{\tau }\bar{Q}_{R}(f_R,f_R)(w,t) \end{aligned} \end{aligned}$$

where $K(\cdot ,\cdot )$ is defined in (3) and the collision-like operators $\bar{Q}_J(\cdot ,\cdot )$, $J\in \mathcal C$, are derived in Sect. 2.2. The system of kinetic equations (14) is further complemented by no-flux boundary conditions at $w = \pm 1$ and contains the information on the spreading of the epidemic in terms of the distribution of opinions of a population of agents.

Integrating model (2) with respect to the *w* variable and recalling that if the interaction function is symmetric, the Fokker–Planck operators are mass and momentum preserving in the presence of no-flux boundary conditions coherently with what we observed for the microscopic binary scheme (6). Hence, we obtain the evolution of mass fractions $\rho _J$, $J \in \mathcal C$,

15 $$\begin{aligned} \begin{aligned} \dfrac{\hbox {d}}{\hbox {d}t} \rho _S(t)&= - \dfrac{\beta }{4} \left( 1-m_I - m_S + m_Sm_I\right) \rho _S\rho _I, \\ \dfrac{\hbox {d}}{\hbox {d}t} \rho _E(t)&= \dfrac{\beta }{4} \left( 1-m_I - m_S + m_Sm_I\right) \rho _S\rho _I - \sigma _E \rho _E, \\ \dfrac{\hbox {d}}{\hbox {d}t} \rho _I (t)&= \sigma _E \rho _E - \gamma \rho _I, \\ \dfrac{\hbox {d}}{\hbox {d}t} \rho _R(t)&= \gamma \rho _I, \end{aligned} \end{aligned}$$

where we observe that $(1-m_I-m_S + m_Sm_I )\rho _S\rho _I= (1-m_I)(1-m_S)\rho _S\rho _I\ge 0$ since $\rho _Im_I,\rho _Sm_S \in [-1,1]$. Unlike the classical SEIR model, the system for the evolution of mass fractions in (15) is not closed since the evolution of $\rho _J$, $\rho _J \in \mathcal C$ depends on the evolution of the local mean opinions $m_J$, $J \in \mathcal C$. The closure of system (15) may be formally obtained by resorting to a limit procedure. The main idea is to observe that the typical time scale of the opinion dynamics is faster than the one of the epidemic, and therefore $\tau \ll 1$. Consequently, for small values of $\tau$ the opinion distribution of the *J*th compartment reaches its local beta-type equilibrium with a mass fraction $\rho _J$ and local mean opinion $m_J$ as verified in Sect. 2.2. In particular, we observe exponential convergence of the derived Fokker–Planck equation (11) towards the local Maxwellian parameterized by the conserved quantities, i.e. $\rho _J$ and $m_J$, see Furioli et al. (2019). We highlight that this assumption is coherent with what stated in the work Poletti et al. (2009) since epidemic transmission is generally slower than the propagation of information.

Hence, to get the evolution of mean values we can multiply by *w* and integrate (14) to get system

$$\begin{aligned} \begin{aligned} \dfrac{\hbox {d}}{\hbox {d}t}(\rho _S(t)m_S(t))&= - \dfrac{\beta }{4}\rho _I(1-m_I)\int _{-1}^1 w(1-w)f_S(w,t)\hbox {d}w, \\ \dfrac{\hbox {d}}{\hbox {d}t}(\rho _E(t)m_E(t))&= \dfrac{\beta }{4}\rho _I(1-m_I)\int _{-1}^1 w(1-w)f_S(w,t)\hbox {d}w - \sigma _E m_E\rho _E, \\ \dfrac{\hbox {d}}{\hbox {d}t}(\rho _I(t)m_I(t))&= \sigma _E m_E\rho _E - \gamma m_I\rho _I, \\ \dfrac{\hbox {d}}{\hbox {d}t}(\rho _R(t)m_R(t))&= \gamma m_I\rho _I, \end{aligned} \end{aligned}$$

which now depends on the second-order moment, making this system not closed. It is now possible to close this expression by using the energy of the beta-type local equilibrium distribution as in (13). We have

16 $$\begin{aligned} m_{2,J} =\rho _J \dfrac{\nu _J + 2m_J^2}{2+\nu _J } , \end{aligned}$$

where $\nu _S = \sigma ^2/\lambda _S$ and $m_J$ is the local mean opinion in the *J*th compartment (1)

Hence, we have

$$\begin{aligned} \dfrac{\hbox {d}}{\hbox {d}t}(\rho _S(t)m_S(t)) = -\dfrac{\beta }{4}(1-m_I)\rho _I\rho _S\left( m_S - \dfrac{\nu _S + 2m_S^2}{2+\nu _S} \right) \end{aligned}$$

which gives

$$\begin{aligned} \rho _S(t)\dfrac{\hbox {d}}{\hbox {d}t}m_S(t) = -\dfrac{\beta }{4}(1-m_I)\rho _I\rho _S\left( m_S - \dfrac{\nu _S + 2m_S^2}{2+\nu _S} \right) - m_S\dfrac{\hbox {d}}{\hbox {d}t}\rho _S \end{aligned}$$

where the time evolution of the fraction $\rho _S$ has been derived in the first equation of (15). The evolution of the local mean $m_S$ is therefore given by

$$\begin{aligned} \dfrac{\hbox {d}}{\hbox {d}t}m_S(t) = \dfrac{\beta }{4}(1-m_I)\rho _I \left[ \dfrac{\nu _S+2m_S^2}{2+\nu _S} - m_S^2 \right] . \end{aligned}$$

We may apply an analogous procedure for the remaining local mean values in the compartments of exposed, infected and recovered to obtain

17 $$\begin{aligned} \begin{aligned} \dfrac{\hbox {d}}{\hbox {d}t} m_S(t)&= \dfrac{\beta }{4}\dfrac{\nu _S}{2+\nu _S}(1-m_I)\rho _I \left[ 1 - m_S^2 \right] . \\ \dfrac{\hbox {d}}{\hbox {d}t}m_E(t)&= \dfrac{\beta }{4}\dfrac{\rho _S\rho _I}{\rho _E}(1-m_I)\left[ m_S - \left( \dfrac{\nu _S+2m_S^2}{2+\nu _S} \right) - m_E(1-m_S) \right] \\ \dfrac{\hbox {d}}{\hbox {d}t} m_I(t)&= \sigma _E \dfrac{\rho _E}{\rho _I} \left( m_E-m_I\right) \\ \dfrac{\hbox {d}}{\hbox {d}t}m_R(t)&= \gamma \dfrac{\rho _I}{\rho _R}\left( m_I - m_R\right) . \end{aligned} \end{aligned}$$

#### Remark 2

In the case of consensus of the susceptible agents, i.e. for $\nu _S \rightarrow 0^+$, we can observe that $\frac{\hbox {d}}{\hbox {d}t}m_S(t) = 0$ which leads to $m_S(t) = m_S(0)$ for all $t \ge 0$. The spread of the infection therefore depends only on the protective behaviour of the agents on the compartment $I \in \mathcal C$. Furthermore, the trajectory of the second equation is decreasing in time since

$$\begin{aligned} \dfrac{\hbox {d}}{\hbox {d}t} m_E(t) = -\dfrac{\beta }{4}(1-m_I)\rho _I (1-m_S)\rho _S \dfrac{m_E}{\rho _E}, \end{aligned}$$

and $$\dfrac{\beta }{4}(1-m_I)\rho _I (1-m_S)\rho _S/\rho _E\ge 0$$.

#### Remark 3

If the local incidence rate $K(f_S,f_I)$ in (3) is such that $\kappa (w,w_*)\equiv \beta >0$ than we easily observe that the evolution of mass fractions are decoupled with the local mean opinions since in this case integrating (2) we get

$$\begin{aligned} \begin{aligned} \dfrac{\hbox {d}}{\hbox {d}t} \int _{-1}^1 f_S(w,t)\hbox {d}w&= -\beta \int _{-1}^1 f_S(w,t)\hbox {d}w \int _{-1}^1 f_I(w,t)\hbox {d}w, \\ \dfrac{\hbox {d}}{\hbox {d}t} \int _{-1}^1 f_E(w,t)\hbox {d}w&= \beta \int _{-1}^1 f_S(w,t)\hbox {d}w \int _{-1}^1 f_I(w,t)\hbox {d}w - \sigma _E \int _{-1}^1 f_E(w,t)\hbox {d}w,\\ \dfrac{\hbox {d}}{\hbox {d}t} \int _{-1}^1 f_I(w,t)\hbox {d}w&= \sigma _E \int _{-1}^1 f_E(w,t)\hbox {d}w - \gamma \int _{-1}^1 f_I(w,t)\hbox {d}w, \\ \dfrac{\hbox {d}}{\hbox {d}t} \int _{-1}^1 f_R(w,t)\hbox {d}w&= \gamma \int _{-1}^1 f_I(w,t)\hbox {d}w. \end{aligned} \end{aligned}$$

Therefore, model (2) for constant $\kappa (w,w_*)\equiv \beta$ reduces to the classical SEIR compartmental model.

#### Remark 4

In the case of non-symmetric interaction function $P(w,w_*)$, the system of macroscopic equations loses the information on the evolution of the mean values. A possible prototype of non-symmetric *P* proposed in Pareschi et al. (2019) is the linear perturbation of a constant, i.e. $P(w,w_*) = P(w_*) = pw_*+q$, $q \in [0,1]$ and $|p| \le \min \{q,1-q\}$. In this case, in Pareschi et al. (2019) it is shown that the mean opinion is not conserved and that the asymptotic distribution functions are given by a Dirac delta distribution $\delta (w-1)$ if $p>0$ or by a Dirac delta $\delta (w+1)$ if $p<0$.

### The Macroscopic Model with Saturated Incidence Rate

It is not restrictive to suppose that infected agents possess enforced situational awareness. For this reasons, we may consider the case in which $m_I(t)=\bar{m}_I\in (0,1)$. From the first equation of (17), we get

$$\begin{aligned} \dfrac{\hbox {d}}{\hbox {d}t}m_S(t) = \dfrac{\beta }{4}\rho _I(t)(1-\bar{m}_I)\dfrac{\nu _S }{2+\nu _S} \left[ 1- m_S^2(t)\right] \end{aligned}$$

with initial condition $m_S(0) = m_S^0 \in [-1,1]$. In particular, if $m_S^0 = \pm 1$, then $m_S(t) = m_S^0$ for all $t\ge 0$; otherwise, if $-1<m_S^0<1$, we get

18 $$\begin{aligned} m_S(t) = \dfrac{\exp \{2 \int _0^t J(\rho _I(s))\hbox {d}s\} - \exp \{C_0\}}{\exp \{C_0\} + \exp \{2 \int _0^t J(\rho _I(s))\hbox {d}s\}}, \end{aligned}$$

with $C_0 = \log \frac{1-m_S^0}{1+m_S^0}$ and $J(\rho _I(s)) = \frac{\beta }{4} \frac{\nu _S}{2+\nu _S}(1-\bar{m}_I)\rho _I(s)\ge 0$. We may easily observe that from (18) we have $m_S(t) \in (-1,1)$ for all $t\ge 0$.

Hence, plugging (18) into the system for the mass fractions (15) we get

19 $$\begin{aligned} \begin{aligned} \dfrac{\hbox {d}}{\hbox {d}t}\rho _S(t)&= -\bar{\beta }H(t,\rho _I)\rho _S(t)\rho _I(t), \\ \dfrac{\hbox {d}}{\hbox {d}t}\rho _E(t)&= \bar{\beta }H(t,\rho _I)\rho _S(t)\rho _I(t) - \sigma _E\rho _E,\\ \dfrac{\hbox {d}}{\hbox {d}t}\rho _I(t)&= \sigma _E\rho _E - \gamma \rho _I,\\ \dfrac{\hbox {d}}{\hbox {d}t}\rho _R(t)&= \gamma \rho _I \end{aligned} \end{aligned}$$

where

$$\begin{aligned} \bar{\beta }H(t,\rho _I) = \bar{\beta }\left( 1-\dfrac{e^{2\int _0^tJ(\rho _I(s))\hbox {d}s}-\hbox {e}^{C_0}}{e^{2\int _0^tJ(\rho _I(s))\hbox {d}s}+\hbox {e}^{C_0}}\right) \in (0,1), \end{aligned}$$

and $\bar{\beta }= \frac{\beta }{4} (1-\bar{m}_I)$. In this case, model (19) is a generalization of classical models with saturated incidence rate, see Capasso and Serio (1978) and Korobeinikov and Maini (2005). In this setting, we derive the basic reproduction number by defining

$$\begin{aligned} D(\rho _S,\rho _I) = \bar{\beta }H(t,\rho _I)\rho _S\rho _I, \end{aligned}$$

and the function $D(\rho _S,\rho _I)$ is such that

$$\begin{aligned} \dfrac{\partial D(\rho _S,\rho _I)}{\partial \rho _S}>0, \qquad \dfrac{\partial D(\rho _S,\rho _I)}{\partial \rho _I} >0 \end{aligned}$$

and $D(\rho _S,\rho _I)$ is concave since $\frac{\partial ^2}{\partial \rho _I^2}D(\rho _S,\rho _I)\le 0$ for all $\rho _S,\rho _I>0$. Hence, the basic reproduction number $R_0$ of the model is given by

$$\begin{aligned} R_0 = \dfrac{1}{\gamma }\lim _{\rho _I\rightarrow 0, \rho _S\rightarrow 1} \dfrac{\partial D(\rho _S,\rho _I)}{\partial \rho _I} = \dfrac{ \beta (1-\bar{m}_I)}{4\gamma }. \end{aligned}$$

For the computation of the basic reproduction number $R_0$ using the method of next-generation matrix, we refer to Bellomo and Chaplain (2022). The method goes back to Diekmann et al. (1990), and we also refer to Diekmann et al. (2009) for an application to the SEIR model.

## Numerical Examples

In this section, we present several numerical examples to show the consistency of the proposed approach. Furthermore, we will show the impact of opinion consensus dynamics on observable epidemic quantities based on beta-type equilibrium and on the macroscopic models generated by bounded-confidence-type opinion distributions. The consensus of the population on the adoption of protective measures is capable of reducing the epidemic peak together with the total number of infected agents. Finally, we will investigate numerically the impact of opinion polarization on the defined dynamics.

From the methodological point of view, we will consider classical direct simulation Monte Carlo (DSMC) methods to show how, in the quasi-invariant limit defined in (9), the large time distribution of the Boltzmann-type model (8) is consistent with the one obtained from the reduced complexity Fokker–Planck model (11). In the following, we will first concentrate on the case of interactions leading to a beta distribution of the form (12). As a follow-up question, we will explore the observable effects of nonlinear interaction functions.

Hence, in order to approximate the dynamics of the kinetic SEIR model (2) for small values of $\tau >0$, we resort to classical strong stability preserving schemes combined to recently developed semi-implicit structure-preserving schemes for nonlinear Fokker–Planck equations (Pareschi and Zanella 2018), see also Loy and Zanella (2021) for further applications. These methods are capable of reproducing large time statistical properties of the exact steady state with arbitrary accuracy together with the preservation of the main physical properties of the solution, like positivity and entropy dissipation. Indeed, we highlight how in the present setting the development of DSMC methods would encounter severe time step restrictions depending on the values of $\tau >0$. We point the interested reader to Pareschi and Russo (2001) for a more detailed discussion on the topic.

### Test 1: Large Time Behaviour of Kinetic Opinion Formation Models

In this section, we test the consistency of the quasi-invariant limit to obtain a reduced complexity Fokker–Planck model. In particular, we concentrate on a kinetic model for opinion formation where the binary scheme is given by (5) in the simplified case $P\equiv 1$ and for $D(w) = \sqrt{1-w^2}$. As discussed in Sect. 2.2, for quasi-invariant interactions as in (9) and in the limit $\epsilon \rightarrow 0^+$, the emerging distribution can be computed through the Fokker–Planck model (11) and is given by the beta distribution (12).

We rewrite the Boltzmann-type model (8) as follows:

$$\begin{aligned} \partial _t f_J(w,t) = \dfrac{1}{\tau }\left( Q^+({f_J},{f_J})(w,t) - {f_J}(w,t)\right) , \end{aligned}$$

where $\tau >0$ is a positive constant and

$$\begin{aligned} Q^+({f_J},{f_J})(w,t) = \left\langle \int _{-1}^1 \dfrac{1}{{}^\prime \mathcal J} {f_J}({}^\prime w,t){f_J}({}^\prime w_*,t) \hbox {d}w_* \right\rangle , \end{aligned}$$

where $({}^\prime w, {}^\prime w_*)$ are the pre-interaction opinions generating the post-interaction opinions $(w,w_*)$ according to the binary interaction rule (5) and ${}^\prime \mathcal J$ is the Jacobian of the transformation $({}^\prime w, {}^\prime w_*)\rightarrow (w,w_*)$. To compute the large time numerical solution of the introduced Boltzmann-type model, we consider $N = 10^6$ particles and we assume that $\tau = 1$. The quasi-invariant regime of parameters in (9) is considered for $\epsilon = 10^{-1},10^{-3}$.

**Fig. 3:**
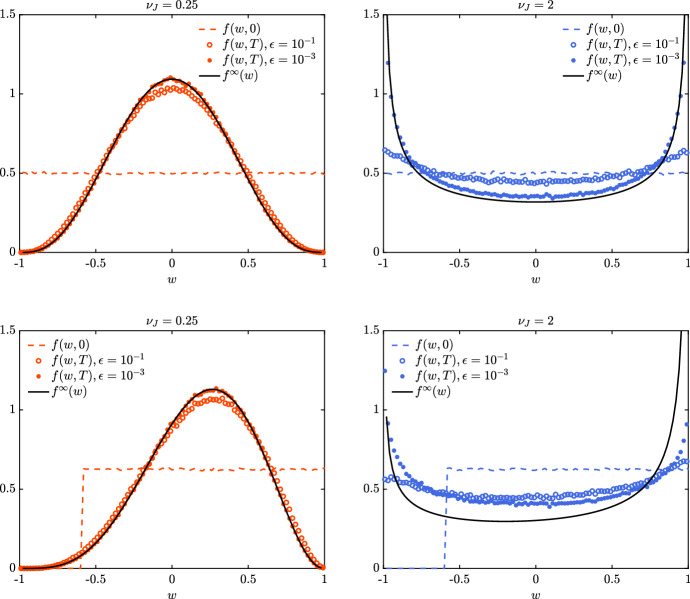
*Test 1*. Comparison between DSMC solution of the Boltzmann-type problem (8) and the beta equilibrium solution of the Fokker–Planck model (11) for several values of $\nu _J = 0.25$ (left column) $\nu _J = 2$ (right column) and choices of the initial distribution. In particular, we considered the choices in (20) (top row) and (21) (bottom row). The DSMC scheme has been implemented with $N = 10^6$ particles over the time frame [0, 5] with $\Delta t = \epsilon = 10^{-1}, 10^{-3}$

In Fig. 3, we depict the densities reconstructed from the DSMC approach with $N = 10^6$ particles at time $T = 5$ and assuming $\Delta t = \epsilon = 10^{-3},10^{-1}$. In the top row, we considered the initial distribution

20 $$\begin{aligned} f_J(w,0) = {\left\{ \begin{array}{ll} \frac{1}{2} &{} w \in [-1,1] \\ 0 &{} w \notin [-1,1] \end{array}\right. } \end{aligned}$$

such that $m_J(0) = \int _{-1}^1 f(w,0)\hbox {d}w = 0$ which is conserved in time. In the bottom row, we consider the initial distribution

21 $$\begin{aligned} f_J(w,0) = {\left\{ \begin{array}{ll} \frac{5}{8} &{} w \in [-0.6,1] \\ 0 &{} w \notin [-0.6,1] \end{array}\right. } \end{aligned}$$

such that $m_J = 0.2$. We further assume that $\lambda _J = 1$ and $\sigma _J^2 = 0.25$ in the left column, whereas $\sigma ^2_J = 2$ in the right column. Hence, under the introduced choice of parameters we have considered $\nu _J = 0.25$ (left column) and $\nu _J = 2$ (right column). The emerging distribution is compared with the beta distribution defined in (12). We may observe how, for decreasing values of $\epsilon \rightarrow 0^+$, we correctly approximate the large time solution of the surrogate Fokker–Planck-type problem.

### Test 2: Consistency of the Macroscopic Limit

In this test, we compare the evolution of mass and local mean of the distributions $f_J$, $J\in \mathcal C$, solution to (2), with the evolution of the obtained macroscopic system (15)–(17).

We are interested in the evolution $f_J(w,t)$, $J\in \mathcal C$, $w \in [-1,1]$, $t \ge 0$ solution to (2) and complemented by the initial condition $f_J(w,0) = f_J^0$. We consider a time discretization of the interval $[0,t_{\text {max}}]$ of size $\Delta t >0$. We denote by $f^n_J(w)$ the approximation of $f_J(w,t^n)$. Hence, we introduce a splitting strategy between the opinion consensus step $f^*_J = \mathcal O_{\Delta t}(f^n_J)$

22 $$\begin{aligned} {\left\{ \begin{array}{ll} \partial _t f_J^* = \dfrac{1}{\tau } \bar{Q}_J(f_J^*,f_J^*), \\ f_J^*(w,0) = f_J^n(w), \qquad J \in \mathcal C \end{array}\right. } \end{aligned}$$

and the epidemiological step $$f^{**}_J = \mathcal E_{\Delta t}(f^{**}_J)$$

23 $$\begin{aligned} {\left\{ \begin{array}{ll} \partial _t f_S^{**} = -f_S^{**}(1-w)\rho _I^{**}(1-m_I^{**}) \\ \partial _t f_E^{**} = f_S^{**}(1-w)\rho _I^{**}(1-m_I^{**}) - \sigma _E f_E^{**} \\ \partial _t f_I^{**} = \sigma _E f_E^{**} - \gamma f_I^{**} \\ \partial _t f_R^{**} = \gamma f_I^{**}, \\ f^{**}_J(w,0) = f^*_J(w,\Delta t). \end{array}\right. } \end{aligned}$$

The operator $\bar{Q}_J(\cdot ,\cdot )$ in (22) has been defined in (11) together with no-flux boundary conditions. Hence, the solution at time $t^{n+1}$ is given by the combination of the two described steps. In particular, a first-order splitting strategy corresponds to

$$\begin{aligned} f_J^{n+1}(w) = \mathcal E_{\Delta t}(\mathcal O_{\Delta t}(f_J^n(w))), \end{aligned}$$

whereas the second-order Strang splitting method is obtained as

$$\begin{aligned} f_J^{n+1}(w) = \mathcal E_{\Delta t/2}(\mathcal O_{\Delta t}(\mathcal E_{\Delta t/2}(f_J^n(w)))), \end{aligned}$$

for all $J \in \mathcal C$. The opinion consensus step (22) is solved by means of a second-order semi-implicit structure-preserving (SP) method for Fokker–Planck equations, see Pareschi and Zanella (2018). The integration of the epidemiological step (23) is performed with an RK4 method. In the following, we will adopt a Strang splitting approach.

We consider the following artificial parameters characterizing the epidemiological dynamics $\beta = 0.4$, $\sigma _E = 1/2$, $\gamma = 1/12$. These values are strongly dependent on the infectious disease under investigation. We highlight that, without having the intention to use real data for the calibration of the presented model, these values are coherent with several recent works for the COVID-19 pandemic (Albi et al. 2022; Buonomo and Della Marca 2020; Dimarco et al. 2022).

### Test 2a: Equilibrium Closure

In this test, we assume a constant interaction function $P(\cdot ,\cdot ) \equiv 1$ such that the Fokker–Planck model is characterized by a beta equilibrium distribution (12) as shown in Sect. 2.2. To define the initial condition, we introduce the distributions

$$\begin{aligned} g(w) = {\left\{ \begin{array}{ll} 1 &{} w \in [-1,0] \\ 0 &{} \text {elsewhere}, \end{array}\right. } \qquad h(w)= {\left\{ \begin{array}{ll} 1 &{} w \in [0,1] \\ 0 &{} \text {elsewhere}, \end{array}\right. } \end{aligned}$$

and we consider

24 $$\begin{aligned} \begin{aligned} f_S(w,0) = \rho _S(0)g(w),\qquad f_E(w,0) = \rho _E(0)g(w),\\ f_I(w,0) = \rho _I(0)h(w),\qquad f_R(w,0) = \rho _R(0)h(w), \end{aligned} \end{aligned}$$

with $$\rho _E(0) =\rho _I(0) = \rho _R(0) = 10^{-2}$$ and $$\rho _S = 1-\rho _E(0)-\rho _I(0)-\rho _R(0)$$.

**Fig. 4:**
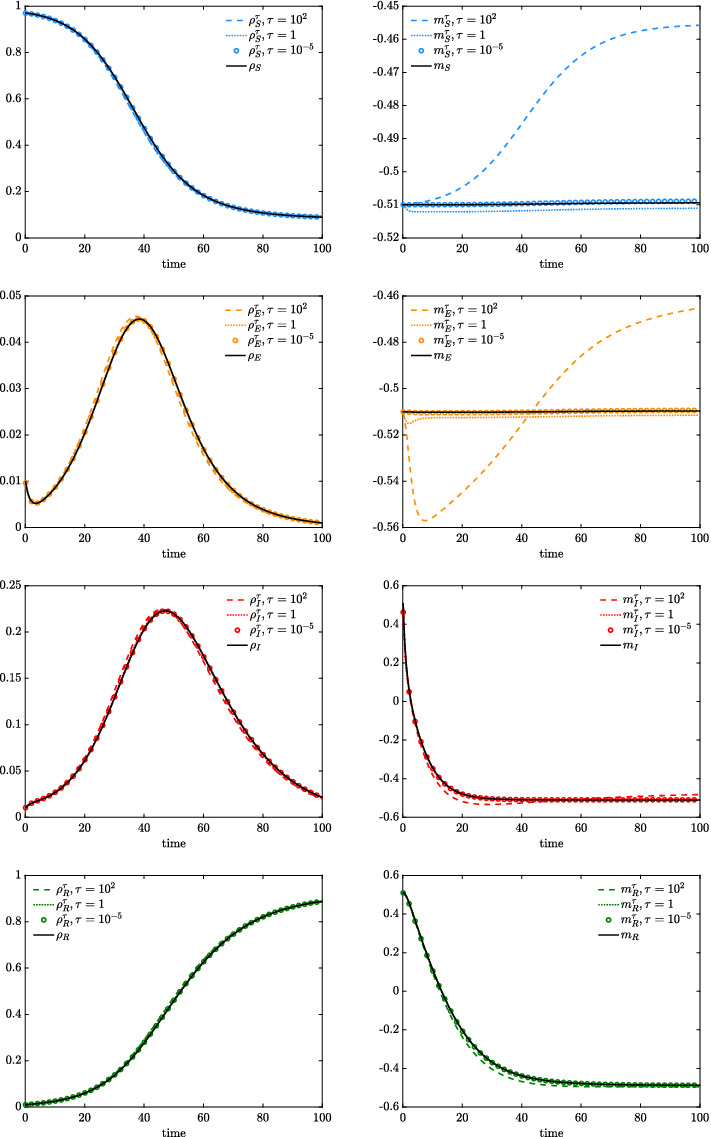
*Test 2a*. Evolution of the macroscopic quantities defined in (15)–(17) and the ones extrapolated from the kinetic model (2) for several values $\tau = 10^{-5},1,10^2$, see (25). Discretization of the domain $[-1,1]$ obtained with $N_w = 201$ gridpoints, discretization of the time frame [0, 100] obtained with $\Delta t = 10^{-1}$. The initial distributions are defined in (24), whereas we fixed $\lambda _J = 1$ and $\sigma ^2_J = 10^{-3}$ for all $J \in \mathcal C$

We solve numerically (22)–(23) over the time frame $[0,t_{\text {max}}]$, and we introduce the grid $w_i \in [-1,1]$ with $w_{i+1}-w_i$, where $\Delta w>0$, $i= 1,\dots ,N_w$. We introduce also a time discretization such that $t^{n} = n\Delta t$, $\Delta t >0$, and $n = 0,\dots ,T$ with $T\Delta t = t_{\text {max}}$. For all the details on the considered numerical scheme, we point the interested reader to Pareschi and Zanella (2018). Hence, for several values of $\tau >0$, we compare the evolution of the computed observable quantities defined as

25 $$\begin{aligned} \rho ^{\tau }_J(t) = \int _{-1}^1 f_J(w,t)\hbox {d}w, \qquad m_J^{\tau }(t) = \dfrac{1}{\rho _J^{\tau }(t)} \int _{-1}^1 w f_J(w,t)\hbox {d}w \end{aligned}$$

with the ones in (15)–(17) whose dynamics have been determined through a suitable kinetic closure in the limit $$\tau \rightarrow 0^+$$. In (25), we highlight the dependence on the scale parameter $$\tau >0$$ through a superscript. It is important to remark that the introduced closure strategy is essentially based on the assumption that opinion dynamics are faster than the ones characterizing the epidemic. Furthermore, we fix as initial values of the coupled system (15)–(17) the values $$\rho _J(0)$$ and $$m_J(0) $$, for all $$J\in \mathcal C$$.

In Fig. 4, we present the evolution of the macroscopic system (15)–(17) and of the observable quantities (25) for several $\tau = 10^{-5},1,100$. The consensus dynamics are characterized by $\lambda _J = 1$, $\sigma _J^2 = 10^{-3}$ for all $J \in \mathcal C$, such that $\nu _S = 10^{-3}$. We can easily observe how, for small values of $\tau \ll 1$, the macroscopic model obtained through a beta-type equilibrium closure is coherent with the evolution of mass and mean of the kinetic model (2).

**Fig. 5:**
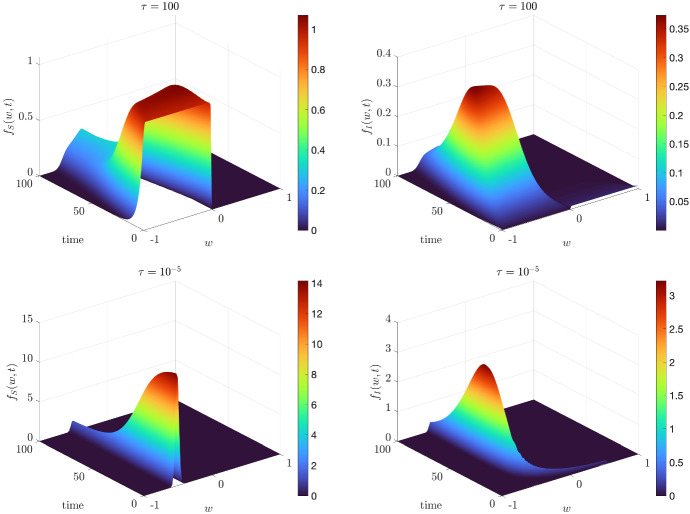
(Color figure online) *Test 2a.* Evolution of the kinetic distributions $f_S$ and $f_I$ over the time interval [0, 100] for $\tau = 100$ (top row) and $\tau = 10^{-5}$ (bottom row). The epidemic dynamics have been characterized by $\beta = 0.4$,$\sigma _E = 1/2$, $\gamma = 1/12$. The solution of the Fokker–Planck step (22) has been performed through a semi-implicit SP scheme over the a grid of $N_w = 201$ nodes and $\Delta t = 10^{-1}$. Initial distributions defined in (24)

In Fig. 5, we show the evolution of the kinetic distributions $f_S(w,t)$ and $f_I(w,t)$ for $t \in [0,100]$. The parameters characterizing the opinion and epidemic dynamics are coherent with the ones chosen for Fig. 4. We may easily observe how for $\tau = 100$ the distributions are far from the beta equilibrium (12), whereas for $\tau = 10^{-5}$ the kinetic distributions $f_J$ are of beta type. Therefore, for small $\tau \ll 1$, the opinion exchanges are faster than the epidemic dynamics and we are allowed to assume a beta-type closure as in (16).

### Test2b: The Bounded Confidence Case

In this test, we consider an interaction function of the form

26 $$\begin{aligned} P(w,w_*) = \chi (|w-w_*|\le \Delta ),\qquad w,w_* \in [-1,1], \end{aligned}$$

where $\chi (\cdot )$ is the indicator function and $\Delta \in [0,2]$ is a confidence threshold parameter above which the agents’ with opinions *w* and $w_*$ do not interact. In the case, $\Delta = 0$ only agents sharing the same opinion interact, whereas for $\Delta = 2$ the interaction function is such that $P(\cdot ,\cdot )\equiv 1$ since $|w-w_*|\le 2$ for all $w,w_* \in [-1,1]$. Bounded confidence-type dynamics have been introduced in Hegselmann and Krause (2002) and have been studied to observe the loss of global consensus. Indeed, for large times, the agents’ opinion forms several clusters whose number and size depend on the parameter $\Delta >0$ and the initial opinions. We highlight that since bounded confidence interactions (26) are symmetric, the mean opinion is preserved in time (Pareschi et al. 2019).

**Fig. 6:**
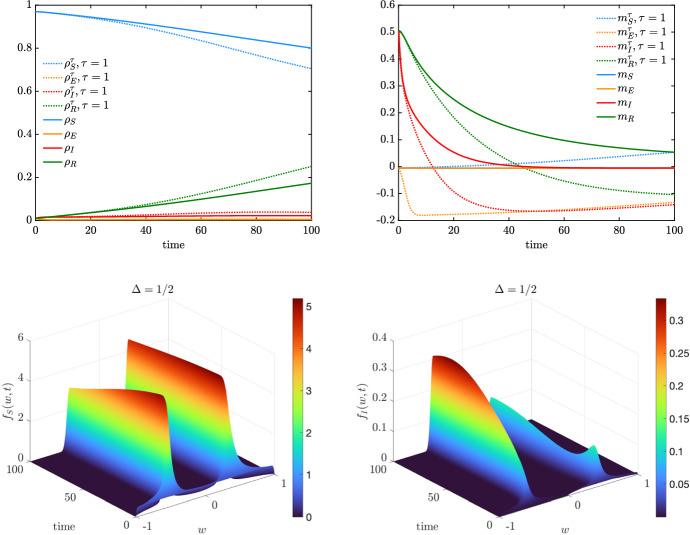
(Color figure online) *Test 2b*. We consider a the bounded confidence interaction function (26) with $\Delta = \frac{1}{2}$. Top row: evolution of mass fractions (left) and mean values (right) for the agents in compartments $\mathcal C$ with $\tau = 1$ and extrapolated from the kinetic model (2) with a Fokker–Planck operator $\bar{Q}(\cdot ,\cdot )(w,t)$ of the form (27). Bottom row: evolution of the kinetic distributions for the compartments $S,I \in \mathcal C$. The solution of the Fokker–Planck step (22) has been performed through a semi-implicit SP scheme over the a grid of $N_w = 201$ gridpoints and $\Delta t = 10^{-1}$. Initial distributions defined in (28)

**Fig. 7:**
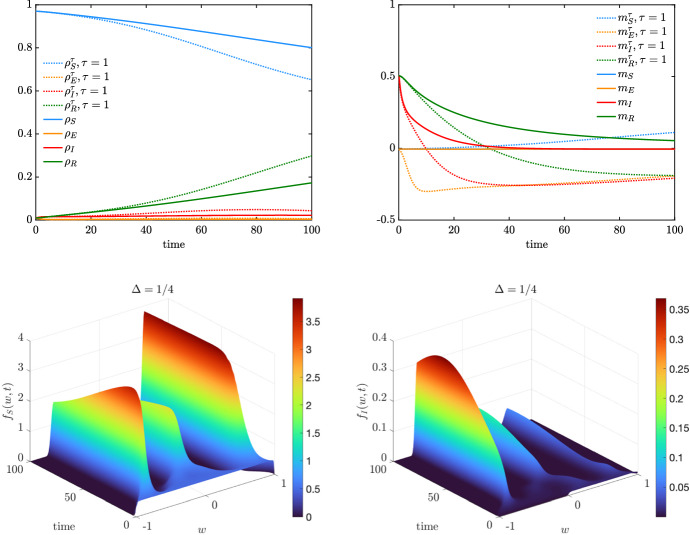
(Color figure online) *Test 2b*. We consider a bounded confidence interaction function (26) with $\Delta = \frac{1}{4}$. Top row: evolution of mass fractions (left) and mean values (right) for the agents in compartments $\mathcal C$ with $\tau = 1$ and extrapolated from the kinetic model (2) with a Fokker–Planck operator $\bar{Q}(\cdot ,\cdot )(w,t)$ of the form (27). Bottom row: evolution of the kinetic distributions for the compartments $S,I \in \mathcal C$. The solution of the Fokker–Planck step (22) has been performed through a semi-implicit SP scheme over the a grid of $N_w = 201$ gridpoints and $\Delta t = 10^{-1}$. Initial distributions defined in (28)

Proceeding as in Sect. 2.2, the Fokker–Planck description of a system of agents in the compartment $J \in \mathcal C$ characterized by bounded confidence interactions is given by the following nonlocal operator

27 $$\begin{aligned} \begin{aligned}&\bar{Q}_J(f_J,f_J)(w,t) \\&\quad = \partial _w \left[ \lambda _J\int _{-1}^1 \chi (|w-w_*|\le \Delta )(w-w_*)f_J(w_*,t)\hbox {d}w_* f_J(w,t) \right. \\&\qquad \left. + \dfrac{\sigma ^2_J}{2}\partial _w (D^2(w)f_J(w,t))\right] \end{aligned} \end{aligned}$$

cf. Remark 1. The equilibrium distribution of the corresponding nonlocal model is not explicitly computable, and the resulting macroscopic models for the evolution of observable quantities may deviate from the ones defined in Sect. 3. Let us consider the densities

$$\begin{aligned} g(w) = {\left\{ \begin{array}{ll} \frac{1}{2} &{} w \in [-1,1] \\ 0 &{} \text {elsewhere}, \end{array}\right. } \qquad h(w) = {\left\{ \begin{array}{ll} 1&{} w \in [0,1] \\ 0 &{} \text {elsewhere} \end{array}\right. } \end{aligned}$$

and we consider the initial distributions

28 $$\begin{aligned} \begin{aligned} f_S(w,0)&= \rho _S(0)g(w),\qquad f_E(w,0) = \rho _E(0)g(w),\\ f_I(w,0)&= \rho _I(0)h(w),\qquad f_R(w,0) = \rho _R(0)h(w) \end{aligned} \end{aligned}$$

with $$\rho _E(0) = 0.01$$, $$\rho _I(0) = 0.01$$, $$\rho _R(0) = 0.01$$ and $$\rho _S(0) = 1-\rho _E(0)-\rho _I(0)-\rho _S(0)$$.

In Fig. 6, we show the evolution of the kinetic distributions $f_S(w,t)$ and $f_I(w,t)$, $t \in [0,100]$ determined by bounded confidence interactions described by the nonlocal Fokker–Planck-type operator (27), with $\Delta = \frac{1}{2}$, $\lambda _J = 1$, and $\sigma ^2_J = 10^{-3}$ for all $J \in \mathcal C$. We may observe how the opinion dynamics lead to two separate clusters centred in $-0.5$ and in 0.5. Furthermore, coherently with the modelling assumptions characterizing the incidence rate $K(f_S,f_I)(w,t)$ in (3) and (4), the cluster with negative opinions looses mass since it is linked to agents with weak protective behaviour. The infection is therefore propagated to these agents and the kinetic distribution $f_I(w,0)$ gains mass for $w<0$. We highlight how the approximated equilibrium density is not coherent with a beta distribution. Therefore, the evolution of the macroscopic quantities cannot be obtained through a classical closure method and we need to solve the full kinetic model (Fig. 7).

### Test 2c: Infection-Driven Bounded Confidence Model

**Fig. 8:**
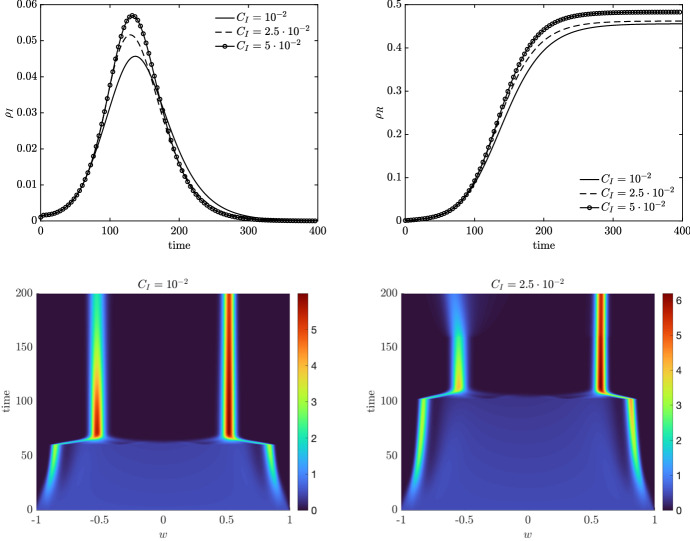
(Color figure online) *Test 2c.* We consider the bounded confidence interaction function (29) with infection-dependent confidence threshold $\Delta (\rho _I)$ defined in (30). Top row: evolution of $\rho _I(t) = \int _{-1}^1 f_I(w,t)\hbox {d}w$ (left) and $\rho _R(t) = \int _{-1}^1 f_R(w,t)\hbox {d}w$ (right) for several values of $C_I = K\times 10^{-2}$ and $K = 1,\frac{5}{2},5$. Bottom row: evolution of the kinetic distributions for the susceptible compartment in the case $C_I = 10^{-2}$ (left) and $C_I = 5 \times 10^{-2}$ (right). The evolution of the kinetic densities has been determined through the semi-implicit SP scheme with $N_w = 201$ gridpoints and $\Delta t = 10^{-1}$. Initial distributions defined in (28) with $\rho _I(0) = \rho _E(0) = \rho _R (0) = 10^{-3}$

We consider in the nonlocal operator (27) the case in which the interaction function depends on the fraction of infected cases $\rho _I(t)$. To this end, we consider the bounded confidence function

29 $$\begin{aligned} P(w,w_*) = \lambda _J \chi (|w-w_*|\le \Delta (\rho _I)), \end{aligned}$$

where $\Delta (\rho _I)$ is a dynamical confidence threshold depending on the epidemic. We further assume that consensus emerges for sufficiently high values of $\rho _I$, mimicking the fact the adoption of a protective behaviour is triggered by the evolution of the epidemic. In particular, we consider

30 $$\begin{aligned} \Delta (\rho _I) = {\left\{ \begin{array}{ll} \Delta _1 &{} \rho _I \le C_I \\ \Delta _2 &{} \rho _I >C_I, \end{array}\right. } \end{aligned}$$

with $\Delta _1< \Delta _2 \in [0,2]$. Therefore, opinion clustering is expected if $\rho _I\le C_I$ and consensus if $\rho _I>C_I$. In Fig. 8, we show the evolution of $\rho _I(t)$ and $\rho _R(t)$ in the case of bounded confidence interactions with infection-driven threshold. The initial conditions are defined in (26). In particular, we consider $\lambda _J = 1$, $\sigma ^2_J = 10^{-3}$ and $\Delta _1 = \frac{1}{10}$ and $\Delta _2 = \frac{1}{2}$, so that that the compromise propensity is higher once the cases escalate. To understand the impact of the parameter $C_I$, we consider $C_I = K \times 10^{-2}$ with $K = 1,\frac{5}{2},5$. We may observe how the epidemic peak is reduced for small values of $C_I>0$. At the same time, the number of recovered agents is reduced for small $C_I>0$. We report also the evolution of the kinetic density $f_S(w,t)$, $t \in [0,200]$ determined by model (14) with $\bar{Q}_J(\cdot ,\cdot )$ defined in (27) and infection-driven bounded confidence interaction function (29). We can observe that the introduced dynamics imply a sharp switch in the compromise process whose effects are also observable the population level.

### Test 3: The Impact of Opinion Polarization on the Infection Dynamics

In this test, we exploit the derived macroscopic system of mass fractions and mean opinions (15)–(17) to investigate the relation between opinion polarization and large number of recovered individuals. We recall that, assuming $P\equiv 1$, opinion polarization is observed if $\nu _S>1$, see Sect. 2.2. Hence, we consider two main cases, supposing that the mean agents’ opinions in all the compartments are exactly alike: the case $m_J(0) = -0.5$, meaning that the agents in each compartment have a bias towards weak protective behaviour, and the case $m_J(0) = 0.5$, meaning that all the agents are biased towards protective behaviour.

**Fig. 9:**
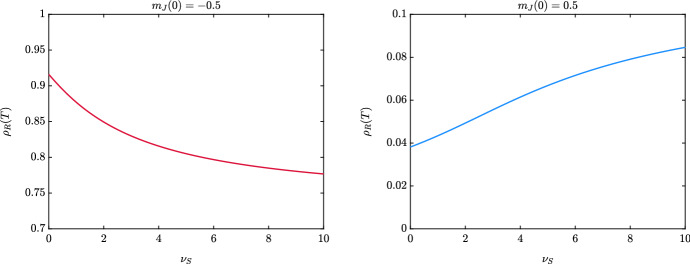
*Test 3*. Impact of the coefficient $\nu _S$ in the large time behaviour of the system (15)–(17) assuming different initial conditions on the mean opinions of the compartments, $m_J(0) = -\,0.5$ (left) and $m_J(0) = 0.5$ (right) for all $J \in \mathcal C$. The epidemiological parameters are the same of the previous tests and fixed as follows $\beta = 0.4$, $\sigma _E =1/2$, $\gamma = 1/12$. Furthermore, we fixed $\rho _E(0 ) = \rho _I(0) = \rho _R(0) = 0.01$ and $\rho _S(0) = 1-\rho _E(0)-\rho _I(0)-\rho _R(0)$. The system of ODEs is solved through RK4 over a time interval [0, 300] with $\Delta t = 10^{-2}$

In Fig. 9, we present the large time mass fractions of recovered individuals $\rho _R(T)$ obtained as solution to (15)–(17) over the time interval [0, *T*], $T = 300$, $\Delta t = 10^{-2}$, where we fixed the value $\nu _S \in [0,10]$. In the left figure we consider the case $m_J(0) = -0.5$, whereas in the right figure we consider the case $m_J(0) = 0.5$. We can observe how the effect of opinion polarization strongly depends on the macroscopic initial opinion of the population on protective behaviour. In details, if the mean opinion is biased towards the adoption of protective behaviour, i.e. $m_J(0)=0.5$, large values of $\nu _S$ trigger an increasing number of recovered individuals, meaning that the infection has a stronger effect on the society in the presence of polarized opinions.

On the other hand, if the initial opinion of the population is biased towards the rejection of protective behaviour, i.e. $m_J(0) = -0.5$, opinion polarization is a factor that can dampen the asymptotic number of recovered individuals. Indeed, opinion polarization in this case pushes a fraction of the population towards the two extreme positions and a fraction of agents will stick towards a maximal protective behaviour.

## Conclusion

In this work, we considered the effects of opinion polarization on epidemic dynamics. We exploit the formalism of kinetic theory for multiagent system where a compartmentalization of the total number of agents is coupled with their opinion evolution. Kinetic models for opinion formation have been developed in detail and are capable of determining minimal conditions for which we can observe polarization of opinions, i.e. the divergence of opinions with respect to a neutral centre. Agents’ opinions on the adoption of protective behaviour during epidemics are a central aspects for the collective compliance with non-pharmaceutical interventions. Thanks to classical methods of kinetic theory, we derived a system of equations that describe the evolution in time of observable quantities that are conserved during the opinion formation process. In particular, considering sufficiently simple interaction functions and local diffusion functions, we get a second-order system of equations for the evolution of mass fractions and mean opinions. This macroscopic system takes into account the social heterogeneities of agents in terms of their opinions and is derived from microscopic dynamics in a SEIR compartmentalization. Thanks to recently developed structure-preserving numerical methods, we showed the consistency of the approach by comparing the system of kinetic equations with the set of macroscopic equations. Furthermore, we analysed more complex interaction functions based on confidence thresholds. The effects of opinion polarization on the asymptotic number of recovered are measured and strongly depend on the initial mean opinion of the population. Indeed, if a positive bias towards protective behaviour is observed, opinion polarization is capable of worsening the infection, whereas if the population tends to reject protective mechanisms, opinion polarization may dampen the total number of infectious agents. Future works will regard more complex opinion formation processes based on leader–follower dynamics and dynamics opinion networks. In future works, we will tackle the calibration of the introduced modelling approach and possible opinion control strategy to prevent the epidemic outbreak.

## Data Availability

The data sets generated during the current study are available from the corresponding author on reasonable request.
